# Editorial: Environmental determinants of cardiovascular health: interactions with lifestyle and socioeconomic factors

**DOI:** 10.3389/fcvm.2026.1890600

**Published:** 2026-06-16

**Authors:** Iván Cavero-Redondo, Alicia Saz-Lara

**Affiliations:** CarVasCare Research Group, Instituto de Biomedicina and Facultad de Enfermería de Cuenca, Universidad de Castilla-La Mancha, Cuenca, Spain

**Keywords:** cardiovascular health, enviromental exposure, health prevention, lifestyle behavior, socieconomic factors

Cardiovascular diseases (CVDs) remain the leading cause of morbidity and mortality worldwide and represent a major and persistent challenge for public health systems (1). In recent years, the scientific landscape has progressively moved beyond a reductionist view focused solely on individual risk factors toward a more comprehensive framework that integrates environmental exposures, lifestyle behaviors, and socioeconomic conditions (2). Within this evolving paradigm, the present research topic was designed to advance the understanding of how these dimensions interact to influence cardiovascular health across populations (3).

The contributions included in this collection consistently highlight that environmental determinants play a critical role in shaping cardiovascular risk, not in isolation but through complex and often synergistic interactions with behavioral and structural factors (4). A substantial body of epidemiological evidence underscores the impact of air pollution as a key environmental driver of cardiovascular morbidity (5). For instance, Liu et al. demonstrated that short-term exposure to extreme concentrations of pollutants such as nitrogen dioxide, sulfur dioxide, and particulate matter was significantly associated with increased coronary heart disease hospitalizations in Henan Province, with marked heterogeneity according to sex and age groups (6). These findings further support the role of acute environmental exposure as a trigger of cardiovascular events in susceptible and vulnerable populations (7, 8).

Beyond the acute cardiovascular effects of air pollution, the mechanistic and synthesis studies included in this research topic provide additional insight into the biological pathways linking environmental exposure to cardiovascular risk (9). In addition, Hamzavi et al. conducted a systematic review and meta-analysis and demonstrated that exposure to persistent organic pollutants, particularly polychlorinated biphenyls (PCBs), was positively associated with hypertension risk, especially for dioxin-like PCB congeners (10). These findings reinforce the mechanistic relevance of oxidative stress and vascular inflammation in environmentally mediated cardiovascular disease (10). This line of research strengthens the conceptualization of environmental exposures as upstream determinants influencing intermediate cardiovascular risk factors (11).

Several studies on this topic also address the global burden attributable to specific environmental exposures, offering valuable perspectives on long-term trends and inequalities (12). Similarly, Deng et al. reported a substantial increase in the global burden of atrial fibrillation and flutter attributable to lead exposure between 1990 and 2021, particularly among older adults and populations from low-SDI regions. Their projections further suggest that the cardiovascular consequences of environmental lead exposure may continue to increase in the absence of stronger mitigation policies (13). These findings highlight the persistent nature of environmental health disparities and the need for context-sensitive policy responses (14).

Broader epidemiological analyses included in this research topic further illustrate the interactions between environmental risk factors, demographic aging, and cardiovascular burden across different populations and socioeconomic contexts. Xu et al. reported ambient particulate matter pollution, elevated systolic blood pressure, and smoking as major contributors to the increasing burden of ischemic heart disease in China (15). Similarly, Zhang et al. demonstrated substantial increases in the incidence and disability-adjusted life years attributable to lower extremity peripheral arterial disease, whereas Yan et al. highlighted persistent disparities in atherosclerotic cardiovascular disease burden among working-age populations across SDI regions. In older populations, Ma et al. reported that although age-standardized mortality rates related to aortic aneurysms have declined globally, the absolute burden continues to increase because of population aging and sustained exposure to modifiable risk factors, particularly smoking. Consistent with these findings, Xue et al. reported substantial increases in the burden of severe heart failure attributable to hypertensive, ischemic, and rheumatic heart diseases, with pronounced inequalities across SDI regions.

Collectively, these findings illustrate how demographic transitions, urbanization, environmental exposure, and structural inequalities interact to shape contemporary cardiovascular disease burden worldwide. Although age-standardized cardiovascular rates have declined in some settings, the absolute number of cases continues to rise, particularly in low- and middle-income regions, emphasizing the importance of integrated prevention strategies and equitable access to healthcare resources (16, 17).

Socioeconomic factors emerge as a cross-cutting dimension throughout the contributions (18). Evidence indicates that disparities in income, education, employment conditions, and access to healthcare significantly influence both exposure to environmental risks and the capacity to mitigate their effects (19). For example, precarious employment has been associated with increased cardiovascular risk and chronic disease burden, suggesting that adverse working conditions may amplify vulnerability through both psychosocial and material pathways (20). Similarly, inequities in clinical outcomes and the management of severe cardiovascular conditions have been documented according to socioeconomic status (21).

In addition to traditional cardiovascular risk factors, Iglesias-Rios et al. demonstrated that precarious employment conditions among agricultural workers were associated with increased chronic disease burden and cardiovascular risk profiles, particularly among women and indigenous workers, emphasizing the importance of occupational and social vulnerability in cardiovascular prevention frameworks.

Socioeconomic determinants also shape access to prevention and treatment. Marcos-Mangas et al. analysed the impact of socioeconomic status among patients with cardiogenic shock and reported persistent inequalities in therapeutic management and outcomes despite treatment within a universal healthcare system, underscoring the relevance of structural determinants in acute cardiovascular care.

Population-based analyses within this collection also explored the interaction between psychosocial, metabolic, and behavioral determinants of hypertension. Song et al. reported significant associations between mental health status, lipid biomarkers, and hypertension risk among oil workers, highlighting the multidimensional nature of cardiovascular risk in occupational settings. Similarly, Senitan et al. characterized the epidemiological profile of hypertension in Saudi Arabia and reported strong associations with obesity, smoking status, diabetes status, hypercholesterolemia status, and educational level, reinforcing the importance of population-based prevention strategies targeting modifiable risk factors. These findings collectively emphasize that cardiovascular prevention cannot be approached exclusively from a biomedical perspective but rather requires integrated strategies addressing behavioral, environmental, and structural determinants.

Lifestyle behaviors, including diet, physical activity, and tobacco use, are consistently identified as key mediators linking environmental and socioeconomic exposures with cardiovascular outcomes (22). In this context, Haber et al. emphasized the critical role of lifestyle interventions and pharmacological prevention strategies in reducing premature cardiovascular mortality and highlighted the emerging relevance of precision medicine and artificial intelligence in cardiovascular risk stratification.

Furthermore, Alrida et al. demonstrated the cumulative impact of standard modifiable cardiovascular risk factors (SMuRFs) on disease severity and cardiovascular outcomes among Middle Eastern patients with atherosclerotic cardiovascular disease and a family history of premature cardiovascular disease, reinforcing the importance of integrated preventive approaches across populations.

Another relevant aspect emerging from this collection is the heterogeneity of effects across population subgroups. Differences by sex, age, and geographic region are recurrently observed, suggesting that susceptibility to environmental and lifestyle factors is not uniform (23, 24). For instance, some studies report a greater burden of disease among middle-aged men, while older women may experience a relatively greater impact in later stages of life (25). These findings underscore the importance of incorporating a precision public health perspective that accounts for differential vulnerabilities.

This research topic highlights important sex- and region-specific disparities in cardiovascular health. Data from the MEA-WCVD Registry provided valuable insights into cardiovascular disease patterns among North African women, emphasizing the importance of culturally and regionally tailored approaches for cardiovascular prevention and management (Charfeddine et al.). Together with the previously described socioeconomic and demographic disparities, these findings reinforce the relevance of precision public health approaches capable of addressing heterogeneous cardiovascular vulnerabilities across populations.

Despite the advances reflected in the contributions to this research topic, important gaps remain. In particular, longitudinal studies capable of simultaneously assessing multiple environmental exposures alongside lifestyle and socioeconomic variables are needed to better capture the complexity of real-world conditions. Future research efforts should prioritize integrative approaches that move beyond single-exposure models and consider the cumulative and interactive nature of environmental risks over time.

In this context, emerging research initiatives are beginning to adopt such comprehensive frameworks, aiming to generate high-resolution evidence on how environmental factors, including air quality, climate variability, and access to natural spaces, interact with individual and contextual determinants to influence cardiovascular health. These future studies will be essential for advancing causal inference and informing more targeted and context-specific prevention strategies.

In conclusion, the present research provides a comprehensive and timely contribution to the understanding of the environmental determinants of cardiovascular health. By emphasizing their interaction with lifestyle and socioeconomic factors, the need for integrated, multidisciplinary approaches to reduce the global burden of CVD is highlighted. Advancing this field will require not only continued scientific inquiry but also the translation of evidence into policies that address environmental quality, promote healthy behaviors, and reduce social inequalities in health.

## References

[1] Global Burden of Cardiovascular Diseases and Risks 2023 Collaborators. Global, regional, and national burden of cardiovascular diseases and risk factors in 204 countries and territories, 1990–2023. J Am Coll Cardiol. (2025) 86(22):2167–243. 10.1016/j.jacc.2025.08.015 40990886

[2] Di Renzo L Gualtieri P Frank G Cianci R Caldarelli M Leggeri G. Exploring the exposome Spectrum: unveiling endogenous and exogenous factors in non-communicable chronic diseases. Diseases. (2024) 12(8):176. 10.3390/diseases12080176 39195175 PMC11353379

[3] Suglia SF Hidalgo B Baccarelli AA Cardenas A Damrauer S Johnson A. Improving cardiovascular health through the consideration of social factors in genetics and genomics research: a scientific statement from the American Heart Association. Circ Cardiovasc Qual Outcomes. (2025) 18(5):e000138. 10.1161/HCQ.0000000000000138 40123498 PMC13333083

[4] Nyavor H Obeng-Gyasi E. Bayesian Network modeling of environmental, social, and behavioral determinants of cardiovascular disease risk. Int J Environ Res Public Health. (2025) 22(10):1551. 10.3390/ijerph22101551 41154955 PMC12562918

[5] de Bont J Jaganathan S Dahlquist M Persson Å Stafoggia M Ljungman P. Ambient air pollution and cardiovascular diseases: an umbrella review of systematic reviews and meta-analyses. J Intern Med. (2022) 291(6):779–800. 10.1111/joim.13467 35138681 PMC9310863

[6] Liu S Wang Y Wang L Li X Fei M Dong P. Short-term effects of extreme air pollutant concentrations on coronary heart disease hospitalization in henan province: a time-stratified case-crossover study. Front Cardiovasc Med. (2025) 12:1538788. 10.3389/fcvm.2025.1538788 40342980 PMC12058883

[7] Tian Y Liu H Wu Y Si Y Song J Cao Y. Association between ambient fine particulate pollution and hospital admissions for cause specific cardiovascular disease: time series study in 184 major Chinese cities. Br Med J. (2019) 367:l6572. 10.1136/bmj.l6572 31888884 PMC7190041

[8] Kazi DS Katznelson E Liu CL Al-Roub NM Chaudhary RS Young DE. Climate change and cardiovascular health: a systematic review. JAMA Cardiol. (2024) 9(8):748–57. 10.1001/jamacardio.2024.1321 38865135 PMC11366109

[9] Perry AS Zhang K Murthy VL Choi B Zhao S Gajjar P. Proteomics, human environmental exposure, and cardiometabolic risk. Circ Res. (2024) 135(1):138–54. 10.1161/CIRCRESAHA.124.324559 38662804 PMC11189739

[10] Hamzavi SF Elahi Vahed I Samadi Shams A Nozari F Gamzeh Latava B Mardukhi S. Association between polychlorinated biphenyls and hypertension risk: a systematic review and meta-analysis. Front Cardiovasc Med. (2025) 12:1529431. 10.3389/fcvm.2025.1529431 40313580 PMC12043693

[11] Münzel T Sørensen M Lelieveld J Landrigan PJ Kuntic M Nieuwenhuijsen M. A comprehensive review/expert statement on environmental risk factors of cardiovascular disease. Cardiovasc Res. (2025) 121(11):1653–78. 10.1093/cvr/cvaf119 40795898 PMC12477681

[12] Clark SN Anenberg SC Brauer M. Global burden of disease from environmental factors. Annu Rev Public Health. (2025) 46(1):233–51. 10.1146/annurev-publhealth-071823-105338 39689276 PMC13237490

[13] Deng L Li Q Li H Li B Cheng Z Xiao Y. The burden and trend prediction of atrial fibrillation and flutter associated with lead exposure: insights from the global burden of disease study 2021. Front Cardiovasc Med. (2025) 12:1638747. 10.3389/fcvm.2025.1638747 40873618 PMC12378705

[14] Smith GS Anjum E Francis C Deanes L Acey C. Climate change, environmental disasters, and health inequities: the underlying role of structural inequalities. Curr Environ Health Rep. (2022) 9(1):80–9. 10.1007/s40572-022-00336-w 35338470

[15] Xu S Liu Z Tang M Xu C. Burden, risk factors, and projections of ischemic heart disease in China (1990–2021): findings from the 2021 GBD study. Front Cardiovasc Med. (2025) 12:1549147. 10.3389/fcvm.2025.1549147 40078456 PMC11897495

[16] Mocumbi AO. Cardiovascular health care in low- and middle-income countries. Circulation. (2024) 149(8):557–9. 10.1161/CIRCULATIONAHA.123.065717 38377254

[17] Cacciatore S Mao S Nuñez MV Massaro C Spadafora L Bernardi M. Urban health inequities and healthy longevity: traditional and emerging risk factors across the cities and policy implications. Aging Clin Exp Res. (2025) 37(1):143. 10.1007/s40520-025-03052-1 40332678 PMC12058932

[18] Bammert P Schüttig W Novelli A Iashchenko I Spallek J Blume M. The role of mesolevel characteristics of the health care system and socioeconomic factors on health care use—results of a scoping review. Int J Equity Health. (2024) 23(1):37. 10.1186/s12939-024-02122-6 38395914 PMC10885500

[19] Hajat A Hsia C O'Neill MS. Socioeconomic disparities and air pollution exposure: a global review. Curr Environ Health Rep. (2015) 2(4):440–50. 10.1007/s40572-015-0069-5 26381684 PMC4626327

[20] Gevaert J Mangot-Sala L Almroth M Badarin K Elling DL Jonsson E. Precarious employment in self-employment: a typology and impact on cardiovascular health conditions in Sweden. Soc Sci Med. (2025) 379:118182. 10.1016/j.socscimed.2025.118182 40381287

[21] Schultz WM Kelli HM Lisko JC Varghese T Shen J Sandesara P. Socioeconomic Status and cardiovascular outcomes: challenges and interventions. Circulation. (2018) 137(20):2166–78. 10.1161/CIRCULATIONAHA.117.029652 29760227 PMC5958918

[22] Hu X Knibbs LD Zhou Y Ou Y Dong GH Dong H. The role of lifestyle in the association between long-term ambient air pollution exposure and cardiovascular disease: a national cohort study in China. BMC Med. (2024) 22(1):93. 10.1186/s12916-024-03316-z 38439026 PMC10913402

[23] Zhernakova DV Sinha T Andreu-Sánchez S Prins JR Kurilshikov A Balder JW. Age-dependent sex differences in cardiometabolic risk factors. Nat Cardiovasc Res. (2022) 1(9):844–54. 10.1038/s44161-022-00131-8 39196077 PMC11357998

[24] Wang M Huang Y Song Y Chen J Liu X. Study on environmental and lifestyle factors for the north-south differential of cardiovascular disease in China. Front Public Health. (2021) 9:615152. 10.3389/fpubh.2021.615152 34336751 PMC8322531

[25] Patwardhan V Gil GF Arrieta A Cagney J DeGraw E Herbert ME. Differences across the lifespan between females and males in the top 20 causes of disease burden globally: a systematic analysis of the global burden of disease study 2021. Lancet Public Health. (2024) 9(5):e282–94. 10.1016/S2468-2667(24)00053-7 38702093 PMC11080072

